# Identification and Characterization of Paramyosin from Cyst Wall of Metacercariae Implicated Protective Efficacy against *Clonorchis sinensis* Infection

**DOI:** 10.1371/journal.pone.0033703

**Published:** 2012-03-21

**Authors:** Xiaoyun Wang, Wenjun Chen, Xiaoli Lv, Yanli Tian, Jingtao Men, Xifeng Zhang, Huali Lei, Chenhui Zhou, Fangli Lu, Chi Liang, Xuchu Hu, Jin Xu, Zhongdao Wu, Xuerong Li, Xinbing Yu

**Affiliations:** 1 Department of Parasitology, Zhongshan School of Medicine, Sun Yat-sen University, Guangzhou, Guangdong, People's Republic of China; 2 Key Laboratory for Tropical Diseases Control of Ministry of Education, Sun Yat-sen University, Guangzhou, Guangdong, People's Republic of China; 3 Department of Biological and Pharmaceutical Engineering, Wuhan Polytechnic University, Wuhan, Hubei, People's Republic of China; Universidade Federal de Minas Gerais, Brazil

## Abstract

Human clonorchiasis has been increasingly prevalent in recent years and results in a threat to the public health in epidemic regions, motivating current strategies of vaccines to combat *Clonorchis sinensis* (*C. sinensis*). In this study, we identified *C. sinensis* paramyosin (*Cs*Pmy) from the cyst wall proteins of metacercariae by proteomic approaches and characterized the expressed recombinant pET-26b-*Cs*Pmy protein (101 kDa). Bioinformatics analysis indicated that full-length sequences of paramyosin are conserved in helminthes and numerous B-cell/T-cell epitopes were predicted in amino acid sequence of *Cs*Pmy. Western blot analysis showed that *Cs*Pmy was expressed at four life stages of *C. sinensis*, both cyst wall proteins and soluble tegumental components could be probed by anti-*Cs*Pmy serum. Moreover, immunolocalization results revealed that *Cs*Pmy was specifically localized at cyst wall and excretory bladder of metacercaria, as well as the tegument, oral sucker and vitellarium of adult worm. Both immunoblot and immunolocalization results demonstrated that *Cs*Pmy was highly expressed at the stage of adult worm, metacercariae and cercaria, which could be supported by real-time PCR analysis. Both recombinant protein and nucleic acid of *Cs*Pmy showed strong immunogenicity in rats and induced combined Th1/Th2 immune responses, which were reflected by continuous high level of antibody titers and increased level of IgG1/IgG2a subtypes in serum. In vaccine trials, comparing with control groups, both *Cs*Pmy protein and DNA vaccine exhibited protective effect with significant worm reduction rate of 54.3% (*p*<0.05) and 36.1% (*p*<0.05), respectively. In consistence with immune responses in sera, elevated level of cytokines IFN-γ and IL-4 in splenocytes suggested that *Cs*Pmy could induce combined cellular immunity and humoral immunity in host. Taken together, *Cs*Pmy could be a promising vaccine candidate in the prevention of *C. sinensis* regarding its high immunogenicity and surface localization.

## Introduction

Human clonorchiasis, caused by the liver fluke *Clonorchis sinensis* (*C. sinensis*), has been increasingly prevalent in recent years, resulted from greater consumption of raw freshwater fish containing infective *C. sinensis* metacercariae [1]. Including 15 million afflicted people in China, more than 35 million people globally were infected by this food-borne parasite [2], [3]. Current evidences from experimental and epidemiological investigations have confirmed the association between *C. sinensis* and cholangiocarcinoma [4], [5], [6]. Moreover, chronic infection by the carcinogenic parasite has been regarded to be responsible for other hepatobiliary diseases such as pyogenic cholangitis, cholelithiasis, cholecystitis and hepatic fibrosis [7]. Increasing infection of *C. sinensis* has led to negative socio-economic impact in epidemic regions and resulted in a threat to the public health. Nonetheless, the complicated molecular mechanism involved in liver fluke-associated hepatobiliary diseases remains to be elucidated, motivating current strategies of vaccines to combat *C. sinensis* [8].

The increasing power of mass spectrometry and proteomics of food-borne trematodes has facilitated to directly identify important molecules as vaccine candidates [9]. Compared with secreted proteins and other components of the parasites, tegumental proteins are more important in nutrition ingestion, immune evasion and host-parasite interaction [10], [11]. For instance, tegumental proteins of schistosome have recently been characterized by proteomic approaches and experimental trials, suggesting novel vaccine candidates for combating human blood flukes [12], [13], [14]. Surface proteomes of *Opisthorchis viverrini* also provided a subset of proteins critical for liver fluke survival as well as the etiology of cholangiocarcinoma [15]. However, to date, little information was known about the tegumental proteins of *C. sinensis*, especially the properties of cyst wall proteins of metacercariae that were crucial in protecting juveniles from being injured by external environment [16]. Here we performed proteomic-based identification and characterization of cyst wall proteins, of which paramyosin was one of the abundant component. Paramyosin has been demonstrated as a myofibrillar protein present in numerous invertebrates including helminth parasites [17], [18]. Moreover, previously studies indicated that paramyosin was a multifunctional molecule that involved in both muscle physiological contraction and immunoregulation [19]. As an immunogenic vaccine candidate, paramyosin has been investigated for the protective effect in various of parasites including *Schistosoma japonicum* [20], [21], *Schistosoma mansoni* [22], *Taenia solium* [23] and *Echinococcus granulosus* [24]. Results from vaccine trails made paramyosin a promising vaccine candidate against *C. sinensis* infection.

In the present study, we identified and characterized paramyosin from the cyst wall of *C. sinensis* metacercariae by proteomic approaches. Both immunoblot and immunolocalization results validated that paramyosin was the component of cyst wall proteins. Results from vaccine trails showed that paramyosin had high immunogenicity and conferred protective effect against *C. sinensis* infection, making *C. sinensis* paramyosin (*Cs*Pmy) as a promising vaccine candidate in the control of *C. sinensis*.

## Materials and Methods

### Parasites and animals

*C. sinensis* metacercariae and *C. sinensis* cercarie were isolated from experimentally infected freshwater fish *Ctenopharyngodon idellus* (*C. idellus*) and freshwater snails *Parafossarulus striatulus* (*P. striatulus*) in our laboratory pool [25]. *C. sinensis* adult worms were recovered from infected livers of Sprague-Dawley (SD) rats, which were purchased from animal center of Sun Yat-sen University and raised carefully in accordance with National Institutes of Health on animal care and the ethical guidelines. All experimental procedures were approved by the Animal Care And Use Committee of Sun Yat-sen University (Permit Numbers: SCXK(Guangdong) 2009-0011).

### In vitro excystation of *C. sinensis* metacercariae for cyst wall proteins

Briefly, 10,000 *C. sinensis* metacercariae were isolated from experimentally infected freshwater fish *C. idellus* by digesting the fish muscle with artificial gastric juice (0.2% HCl, 0.6% pepsin, pH 2.0) at 37°C for 2 h. Viability and integrity of metacercariae were assessed under microscope (×100). 0.001% trypsin (Promega, Wisconsin,USA) in physiological saline was employed as excystation stimulus *in vitro*. After activation, cyst wall of metacercariae was immediately collected in RIPA lysis buffer (Amresco, Solon, USA), added with 1 mM Phenylmethanesulfonyl fluoride (PMSF, Sigma, St. Louis, USA). The cyst wall was placed on ice in RIPA lysis buffer and then subjected to centrifugation at 8,000 g at 4°C for 15 min to remove the sediment, 4-fold cold acetone (containing 0.07% β-mercaptoethanol) was added to the supernatant and stored at −20°C overnight. Subsequently, repeated the washing procedures with 4-fold cold acetone and removed the supernatant after centrifugation, then the sediment was treated with 1× loading buffer (50 mM Tris, 2% SDS, pH 6.8) to get cyst wall proteins which were subjected to sodium dodecyl sulfate polyacrylamide gel electrophoresis (SDS-PAGE, 8% gel) followed by Coomassie Blue staining.

### Proteomic identification of *C. sinensis* metacercariae cyst wall proteins by high performance liquid chromatography-tandem mass spectrometry (HPLC-MS/MS)

Gel lanes in SDS-PAGE to be analyzed were excised, about ten visible gel sections were separated and divided into small pieces, all pieces were washed in sterile water and completely destained using destaining solution (25 mM ammonium bicarbonate, 50% acetonitrile). Subsequently, trypsin digestion was performed as described [26]. The reduction step was performed by adding 100 µL of 10 mM DTT (25 mM ammonium bicarbonate) into the samples and incubating at 37°C for 3 h. Protein alkylation was done by adding 100 µL of 55 mM iodoacetamide (25 mM ammonium bicarbonate) and reacted in the dark at 20°C for 30 min. Gel pieces were then treated with 50% acetonitrile and digested with 0.02 µg/µl sequencing grade modified trypsin (Promega) at 37°C overnight. The peptides were then extracted with extraction buffer (67% acetonitrile, 2.5% trifluoroacetic acid) and completely dried in a SpeedVac centrifuge (Thermo Fisher Scientific, Waltham, USA). Dried peptides were analyzed with a Finnigan Surveyor HPLC system coupled online with LTQ-Oribitrap XL (Thermo Fisher Scientific) equipped with a nanospray source. HPLC-MS/MS experiment was carried out at the Institute of Life and Health Engineering and National Engineering Research Center of Genetic Medicine at Jinan University in China. Bioinformatics analysis was performed by inputting the amino acids into the Protein Information Resource (http://pir.georgetown.edu/cgi-bin/batch.pl) and NCBI Database (http://www.ncbi.nlm.nih.gov/). Identified peptides were annotated with predicted names and listed with corresponding database accession numbers.

#### Bioinformatics analysis of *Cs*Pmy

We found three genes (clone numbers: *Cs*020c02/*Cs*032a06/*Cs*046e06) annotated with paramyosin in our *C. sinensis* metacercaria cDNA plasmid library by searching the keyword ‘paramyosin’. We sequenced the corresponding plasmids to get the full-length complete encoding sequence of *Cs*Pmy and then analyzed the nucleotide sequence with BLASTx. The open reading frame (ORF) was found with ORF finder tool in NCBI database (http://www.ncbi.nlm.nih.gov/). Sequence alignment was done by comparing the amino acid sequence of pamamyosin from our laboratory (*C. s-*1, JQ041818) with that of other helminthes including *C. sinensis* from Korea laboratory (*C. s-*2, ABN79674.1), *Paragonimus westermani* (*P. w*, AAY44740.1), *Schistosoma haematobium* (*S. h*, BAF62291.1), *Schistosoma japonicum* (*S. j*, AAA81003.1), *Schistosoma mansoni* (*S. m*, AAA29915.1), *Taenia solium* (*T. s*, AAK58494.1) and *Echinococcus granulosus* (*E. g*, CAA79849.1) using software Vector NTI suite 8.0. Physicochemical properties and conserved domains were predicted with Proteomics tools in ExPaSy web site (http://www.expasy.org/). Both B-cell and T-cell linear epitopes were analyzed by the tools at http://www.cbs.dtu.dk/services/. Nucleotide and amino acid sequences described in the present study have been submitted to GenBank database under the accession number JQ041818.

### Recombinant plasmid construction and purification of *Cs*Pmy protein

The ORF of *Cs*Pmy was amplified by polymerase chain reaction (PCR) from the recombinant plasmid of cDNA library. Specific PCR primers used in the present study were listed in Table S1. The PCR products were purified and firstly cloned into PMD19-T clone vector, and subsequently transformed into *E. coli* DH5α cells. After sequencing, the recombinant plasmid DNA was digested with corresponding restriction enzymes and then the ORF of *Cs*Pmy was subcloned to expression plasmids including prokaryotic expression vector His_6_ tag pET-26b(+) (Qiagen, California, USA) and eukaryotic expression vector pcDNA™-3.1(+), respectively. The expression of the recombinant fusion protein (pET-26b-*Cs*Pmy) in *E. coli* BL21 (DE3) was induced by isopropy-β-D-thiogalactoside (IPTG) at a final concentration of 1 mM at 37°C for 5 h in Luria-Bertani medium (containing 50 µg/ml kanamycin). Lysate of *E. coli* with pET-26b-*Cs*Pmy was collected after centrifugation and treated with ultrasonication to separate the supernatant and sediment. The sediment containing the recombinant fusion protein was washed with Washing buffer (50 mM Tris-HCl, 50 mM NaCl, 1 mM EDTA, 1% Triton X-100, 2 M-urea, pH 8.0) and dissolved with Dissolved buffer (50 mM Tris-HCl, 50 mM NaCl, 1 mM EDTA, 1% Triton X-100, 6 M-urea, pH 8.0) followed by centrifugation. Subsequently, the supernatant was gradiently treated with 5–200 mM imidazole (containing 6 M-urea). After Ni-NTA affinity chromatograph, the purified protein was renatured by gradient-urea dialysis from 4 M to 0 M. Finally, the renatured protein was dialyzed in phosphate buffer saline (PBS), the purity and concentration of pET-26b-*Cs*Pmy were analyzed by 8% SDS-PAGE followed by Coomassie blue staining. Recombinant pcDNA-*Cs*Pmy and empty pcDNA plasmids (in PBS) were isolated from *E. coli* DH5α and the A260/A280 ratio was measured spectrophotometrically for quality determination. The purified recombinant protein and plasmids were stored at −80°C for use.

### Preparation for total worm extracts (TWE), soluble tegumental components and the antiserum of recombinant *Cs*Pmy protein

Briefly, adult worms, metacercariae, cercariae and eggs were crushed to prepare the TWE with the lysis buffer containing 1 mM PMSF (Sigma), the Bradford assay was used to determine the final concentration of TWE. Soluble tegumental components of adult worm were obtained by ProteoExtract Native Membrane Protein Extraction kit (Merck KGaA, Darmstadt, Germany) with the methods previously described [7]. Recombinant pET-26b-*Cs*Pmy was emulsified with complete Freund's adjuvant and subcutaneously injected to SD rats, each animal was given 200 µg recombinant protein for the first injection, and 100 µg recombinant protein emulsified with incomplete Freund's adjuvant was given for the next two boosters at 2-week interval. The rat sera were collected 2 weeks post the last injection and stored at −80°C for use.

### Identification of *Cs*Pmy by SDS-PAGE and Western blot analysis

The recombinant pET-26b-*Cs*Pmy (2 µg/lane), TWE of four life stages (adult worm, metacercaria, cercaria and egg, 10 µg/lane) were resolved by 8% SDS-PAGE and then immobilized onto PVDF membrane. At the same time, cyst wall proteins of metacercaria and soluble tegumental components of adult worm were also immobilized onto PVDF membrane. The membrane was blocked with 5% (*w/v*) skim milk at 4°C overnight, and then incubated with anti-pET-26b-*Cs*Pmy rat serum and naïve rat serum (1∶2000 dilutions in 1% BSA-PBS) at room temperature for 2 h after the washing procedure. Subsequently, the membrane was followed by incubation with rabbit anti-rat IgG HRP-conjugated secondary antibody (1∶2000 dilutions in 1% BSA-PBS, Proteintech Group, Chicago, USA) at room temperature for 1 h. The protein bands were visualized by enhanced chemiluminescence (ECL) method.

### Transcriptional level of *Cs*Pmy at different developmental stages by real-time quantitative PCR (qRT-PCR)

To investigate mRNA expression pattern of *Cs*Pmy at different developmental stages of *C. sinensis* (adult worm, metacercaria, cercaria and egg), we carried out qRT-PCR experiments among the four stages. Total RNA from parasites of four stages were extracted by TRIzol methods (Invitrogen, California, USA) according to the manufacturer's instructions and spectrophotometrically quantitated. Reverse transcription reactions were carried out to get the first-strand cDNA using RT-PCR Kit (TaKaRa, Dalian, PR China) with the same quantity of total RNA as the template (1 µg). β-actin of *C. sinensis* (accession number: EU109284) was used as the transcription control. The primers for *Cs*β-actin amplification were listed in Table S1 and confirmed that they were optimized for amplification efficiency in qRT-PCR experiments. The real-time PCR amplification was performed using the LightCycler480 instrument (Roche, Switzerland) using the SYBR Premix ExTaq Kit (TaKaRa). The PCR amplification program was 95°C for 30 sec, followed by 40 cycles of 95°C for 5 sec and 60°C for 20 sec. The melting curve was performed using a program of 95°C for 30 sec and 65°C for 15 sec. The LightCycler480 software (version 1.5) was used to analyze the data according to the 2^−ΔΔCt^ method [27]. The transcript of egg was employed as the calibrator to evaluate relative expression levels of *Cs*Pmy.

### Immunohistochemical localization of *Cs*Pmy at adult worm and metacercaria

Adult worms and metacercariae of *C. sinensis* were fixed with 4% paraformaldehyde, embedded with paraffin and sliced into 3–5 µm in thick. All sections were dewaxed with dimethylbenzene and treated with 100%, 95%, 85%, and 75% alcohol, respectively. The sections were blocked with normal goat serum overnight at 4°C, and then incubated with anti-pET-26b-*Cs*Pmy rat serum and naïve rat serum at room temperature for 2 h, the serum was diluted at 1∶200 for all sections. After washing three times with PBST (containing 0.1% Tween-20), the sections were incubated with goat anti-rat IgG (1∶400 dilutions in 0.1% BSA-PBST, Alexa Fluor 594, Molecular Probes, California, USA) at room temperature for 1 h in dark and imaged under fluorescence microscope (ZEISS, Goettingen, Germany).

### Vaccination protocols

To explore the protection efficacy of *Cs*Pmy against *C. sinensis* infection, we carried out the preliminary vaccination experiments in rats. Thirty two six-week-aged Sprague Dawley rats were randomly divided into four groups as pET-26b-*Cs*Pmy group, PBS group, pcDNA-*Cs*Pmy group, and pcDNA group, each of which consisted of eight rats. Generally, 200 µg/per rat of recombinant pET-26b-*Cs*Pmy or equivalent volume PBS was subcutaneouly injected with Freund's adjuvant at week 0, week 2, and week 4. 200 µg/per rat of pcDNA-*Cs*Pmy or pcDNA plasmid was injected intramuscularly in quadriceps in the same frequency. All rats were kept under the same conditions until sacrificed, the protection experiments in the present study were carried out blindly.

### Antibody titers and IgG isotype measurement

In order to analyze the immune responses to *Cs*Pmy, we measured the antibody titers of total IgG in immunized sera by enzyme linked immunosorbent assay (ELISA). Briefly, 1 µg/well recombinant pET-26b-*Cs*Pmy protein was coated with coating buffer (0.05 M carbonate-bicarbonate, pH 9.6) and blocked with 5% skimmed milk. After washing procedure, the plate was incubated with different dilutions of the immune sera (week 6) raised by pET-26b-*Cs*Pmy and pcDNA-*Cs*Pmy. Rat sera immunized with PBS and pcDNA were measured under the same conditions as negative controls. Subsequently, HRP-conjugated IgG (1∶20000 dilutions in 0.1% BSA-PBST, Proteintech Group) was used as the secondary antibodies. After 1 h incubation the plate was washed three times with PBST and the reactions were developed by adding 100 µl substrate solution 3, 3′, 5, 5′-Tetramethylbenzidine (TMB, BD biosciences, San Diego, USA). After 5 min incubation in dark, reactions were stopped by adding 50 µl 2 M H_2_SO4 and absorbance was measured at 450 nm. After measuring the antibody titer, to investigate the tendency of circulation antibodies and the profiles of immune responses, we tested the level of total IgG and IgG isotype by diluting the sera (week 2, 4, 6) at 1∶400. IgG (1∶20000 dilutions), IgG1 and IgG2a (1∶1000 dilutions in 0.1% BSA-PBST, Bethyl, Texas, USA) were employed as secondary antibodies.

### Evaluation of vaccine efficacy against challenge

After the measurement of antibody titers at week 6 post immunization, remaining rats (*n* = 6 for each group) were anesthetized with ether and challenged with 100 living *C. sinensis* metacercariae by intragastric administration.

The previously described egg counting method [28] was employed to calculate eggs per gram feces (EPG) from weeks 4 post challenge infection. The experimental rats were sacrificed at week 6 post infection to recover adult worms from livers for worm burden evaluation. Reduction rates in parasite burden were calculated as follows. Worm reduction rate (%) = [(average worm burden of control group–average worm burden of experimental group)/average worm burden of control group]×100%. Egg reduction rate (%) = [(average EPG of control group–average EPG of experimental group)/average EPG of control group]×100%.

### Statistics and software

SPSS version 16.0 software was used in the present study for all statistical analysis. Results for analysis represented mean ± S.D., the recovered worm numbers and EPG in groups were compared by Student's *t*-test, and *p* value of <0.05 was considered significant difference.

## Results

### Microscopical examination of parasites and cyst wall proteins

**Figure 1:**
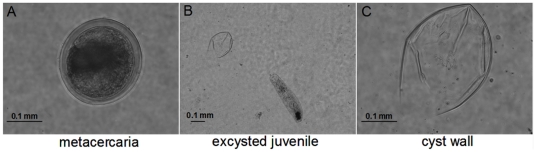
Microscopical examination of *C. sinensis* metacercariae for cyst wall proteins. (A) Intact metacercaria (×400); (B) Excysted juvenile, juvenile immediately moved out (×100); (C) Cyst wall (×400). Bar = 0.1 mm.

Under the microscope, *C. sinensis* metacercariae presented round-shaped appearance with intact cyst wall, with the juveniles inside (Figure 1A). *In vitro* excystation of *C. sinensis* metacercariae was performed by adding 0.001% trypsin to the medium. After activation, most of juveniles immediately moved out (Figure 1B), remaining only the cyst wall (Figure 1C). 500 µg proteins were routinely processed for HPLC-MS/MS. Molecular mass of cyst wall proteins ranged from 11 kDa to 150 kDa (see Table 1). Protein peptides were bioinformatics analyzed, yielding nine proteins with contaminations excluded (see Table 1). Among the matched peptides, paramyosin got high score in match results, with a description of muscle component. The remaining matched proteins were related to protein-protein interactions, motor activity, protein binding, collagen superfamily, cell-surface receptor, and membrane component, respectively. In general, the identified cyst wall proteins could be classified as structural proteins and interaction proteins based on their biological functions.

**Table 1:** Proteins from cyst wall of *C. sinensis* metacercariae identified by HPLC-MS/MS.

Prot_acc	Prot description	Species	Prot_score	Prot_match	Prot_cover	Prot_mass
gi|238658811	paramyosin	*Schistosoma mansoni*	92	6	1.7	131,296
gi|14324125	paramyosin	*Taenia solium*	92	6	4.8	98,840
gi|126116628	paramyosin	*Clonorchis sinensis*	87	17	6.5	99,452
gi|238660770	expressed protein	*Schistosoma mansoni*	60	240	1.5	150,394
gi|156600431	myosin heavy chain	*Clonorchis sinensis*	50	3	8.5	44,118
gi|76154067	SJCHGC06106 protein	*Schistosoma mansoni*	40	5	2.1	50,610
gi|76155574	SJCHGC08294 protein	*Schistosoma mansoni*	27	2	10.2	11,337
gi|256080653	titin	*Schistosoma mansoni*	26	37	3.4	69,682
gi|227284694	stomatin-related	*Schistosoma mansoni*	22	4	1.3	108,702

Prot_acc, protein accession number in databases; Prot description, the most likely matched protein name in the NCBI database. Prot_score, the similarity that peptide matched in the NCBI database. prot_match, the matched peptides in the NCBI database. Prot_cover, the percent of identified amino acids in target amino acid sequence. Prot_mass, molecular mass of matched proteins. Score >50 indicates extensive similarity (*p*<0.05) in the NCBI database.

### Isolating and sequence analysis of *Cs*Pmy sequence

**Figure 2:**
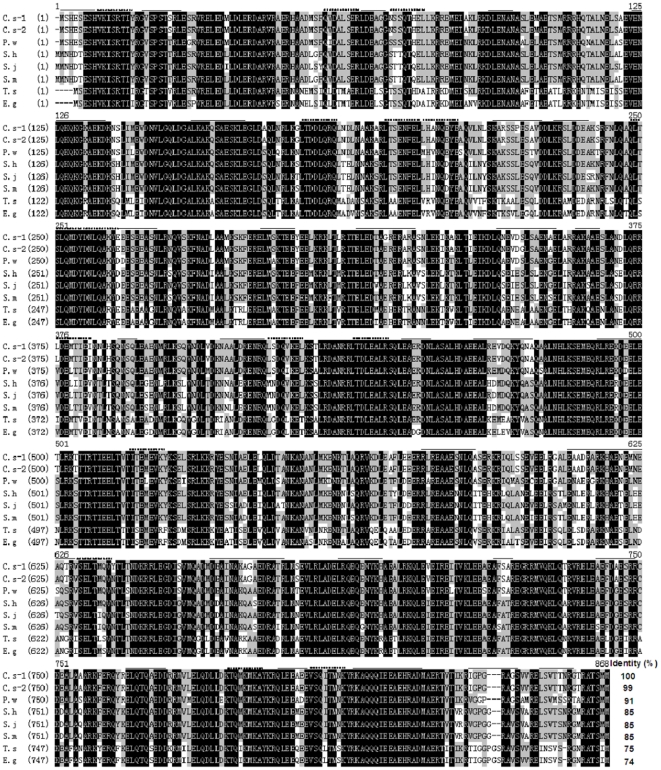
Multiple sequence alignment of deduced amino acid sequence of paramyosin among helminthes. *C. s-*1 (JQ041818) represents the sequence from our *C. sinensis* metacercaria cDNA plasmid library. *C. s-*2 (ABN79674.1) represents the sequence submitted by the laboratory from Korea. *Paragonimus westermani* (*P. w*, AAY44740.1), *Schistosoma haematobium* (*S. h*, BAF62291.1), *Schistosoma japonicum* (*S. j*, AAA81003.1), *Schistosoma mansoni* (*S. m*, AAA29915.1), *Taenia solium* (*T. s*, AAK58494.1) and *Echinococcus granulosus* (*E. g*, CAA79849.1). Amino acids shared among helminthes were indicated in black, high conserved amino acids among helminthes were indicated in gray. B-cell and T-cell linear epitopes were indicated with full lines and dotted lines, respectively.

Three genes annotated with ‘paramyosin’ were isolated from our *C. sinensis* metacercaria cDNA plasmid library, we got the full-length complete encoding sequence of *Cs*Pmy (3465 bp) with an ORF of *Cs*Pmy contained 2595 bp encoding 864 aa (see Figure S1). After comparing the *Cs*Pmy sequences from our metacercaria cDNA library (*C. s-*1) with the sequences submitted by the laboratory from Korea (*C. s-*2), we found that six base pairs and four amino acids were different between nucleotide sequences and amino acid sequences, respectively. However, five identified peptides in HPLC-MS/MS results were all matched with amino acid sequences both in *C. s-*1 and *C. s-*2 with the protein coverage of 6.5% (see Figure S1). Sequence alignment showed that amino acid sequence paramyosin are conserved in helminthes including *C. s-*1, *C. s-*2, *Paragonimus westermani*, *Schistosoma haematobium*, *Schistosoma japonicum*, *Schistosoma mansoni*, *Taenia solium* and *Echinococcus granulosus* (Figure 2) with the identity of 74–99%. 29 B-cell and 15 T-cell linear epitopes were predicted in amino acid sequence of *Cs*Pmy, implying high immunogenicity of this molecule. Bioinformatics analysis showed the predicted molecular mass of recombinant pET-26b-*Cs*Pmy was 101.097 kDa and theoretical isoelectric point was 5.51. The conserved domain of myosin tail was localized at 22 aa-841 aa while no signal peptide or transmembrane region was found in amino acid sequence of *Cs*Pmy.

### Cloning, expression and purification of *Cs*Pmy protein

**Figure 3:**
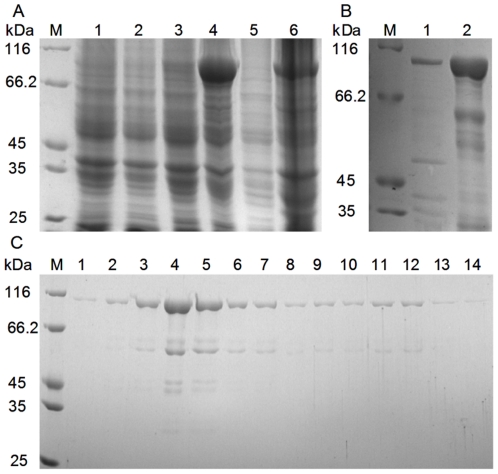
Expression and purification of recombinant pET-26b-*Cs*Pmy identified by 8% SDS-PAGE. (A) Expression of pET-26b-*Cs*Pmy. Protein molecular weight marker (M), lysate of *E. coli* with pET-26b(+) before induction with IPTG (lane 1) and after induction (lane 2), lysate of *E. coli* with pET-26b-*Cs*Pmy before induction with IPTG (lane 3) and after induction (lane 4), supernatant of induced *E. coli* with pET-26b-*Cs*Pmy (lane 5) and sediment (lane 6). (B) Denaturation of inclusion bodies containing pET-26b-*Cs*Pmy. Supernatant collected from inclusion bodies dissolved in 2 M urea (lane 1) and 6 M urea (lane 2). (C) Purification of pET-26b-*Cs*Pmy. Protein eluted with 40 mM imidazole (lane 1–3), 80 mM imidazole (lane 4–8), 100 mM imidazole (lane 9–12), 200 mM imidazole (lane 13–14). Proteins were visualized by Coomassie Blue staining, the protein bands were around 100 kDa.

**Figure 4:**
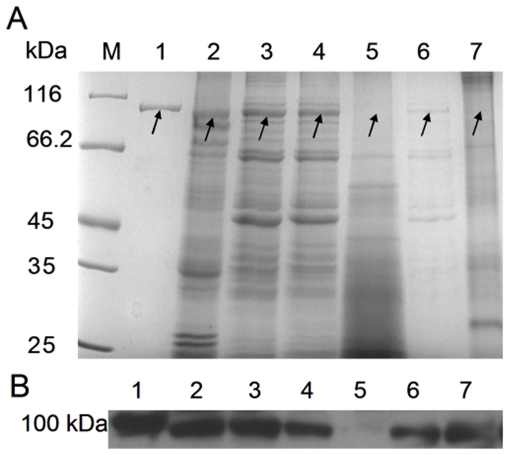
Identification of *Cs*Pmy by SDS-PAGE and Western blot analysis. Protein molecular weight marker (M), purified pET-26b-*Cs*Pmy protein (lane 1), TWE of adult worm (lane 2), TWE of metacercaria (lane 3), TWE of cercaria (lane 4), TWE of egg (lane 5), cyst wall proteins of metacercaria (lane 6), and soluble tegumental components of adult worm (lane 7). (A) 8% SDS-PAGE. (B) Western blot analysis. Corresponding proteins were subjected to 8% SDS-PAGE and immobilized onto the membrane, then the membrane was incubated with anti-pET-26b-*Cs*Pmy rat serum (1∶2000 dilutions) at room temperature for 2 h. Subsequently, the membrane was followed by incubation with rabbit anti-rat IgG HRP-conjugated secondary antibody (1∶2000 dilutions) at room temperature for 1 h. 2 µg of purified pET-26b-*Cs*Pmy protein and 10 µg of TWE were loaded per lane. SDS-PAGE was visualized by Coomassie Blues staining and the protein bands that might be native paramyosin in different life stages were indicated with arrows. Western blot was visualized by ECL method, the detected protein bands were around 100 kDa.

The recombinant pET-26b-*Cs*Pmy protein was overexpressed as inclusion bodies in *E. coli* BL21 (DE3) with a molecular mass around 100 kDa (Figure 3A, lane 4 and 6). The inclusion bodies could be abundantly dissolved with 6 M urea (Figure 3B, lane 2) and eluted with 200 mM imidazole in high purity. After gradient renaturation and concentration, the purified pET-26b-*Cs*Pmy protein was collected (Figure 4A, lane 1). A260/A280 ratio (1.85∼1.9) of pcDNA-*Cs*Pmy and pcDNA plasmids confirmed the high quality of isolated plasmids.

### Identification of *Cs*Pmy by SDS-PAGE and Western blot analysis

Anti-pET-26b-*Cs*Pmy rat serum was used to probe recombinant *Cs*Pmy (Figure 4B, lane 1), TWE of adult worms (Figure 4B, lane 2), TWE of metacercariae (Figure 4B, lane 3), TWE of cercariae (Figure 4B, lane 4) and TWE of eggs (Figure 4B, lane 5). The expression level of *Cs*Pmy at adult worm and metacercaria were higher than that of cercaria and egg. Moreover, HPLC-MS/MS results were validated by Western blot analysis, cyst wall proteins of metacercariae (Figure 4B, lane 6), and soluble tegumental components of adult worms (Figure 4B, lane 7) could be probed by antiserum. In SDS-PAGE, molecular mass of cyst wall proteins were similar to that of the highly expressed proteins in metacercariae, indicating that *Cs*Pmy may be an important component of the cyst wall proteins in metacercariae (Figure 4B, lane 3).

### Transcriptional level of *Cs*Pmy at different developmental stages

**Figure 5:**
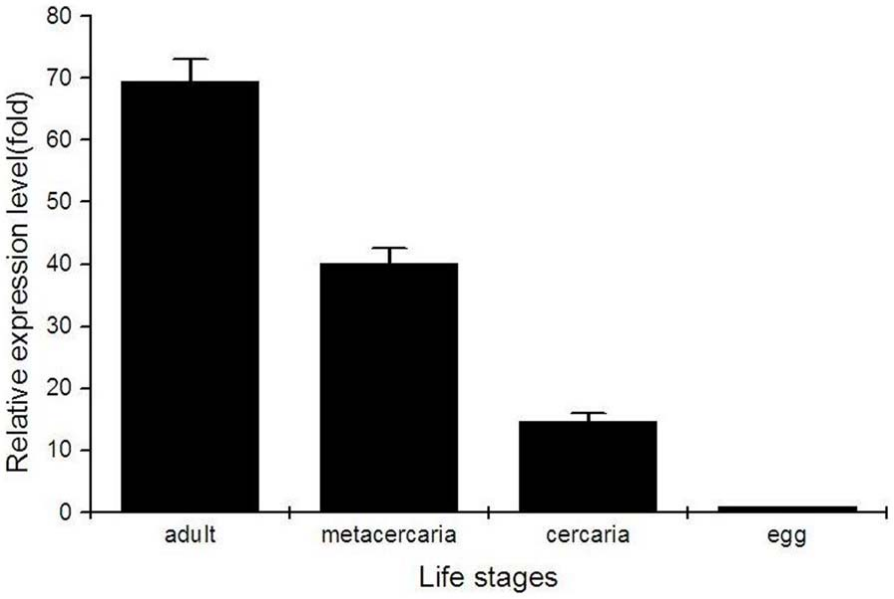
Transcriptional level of *Cs*Pmy at different developmental stages of *C. sinensis* by qRT-PCR experiments. Total RNA from four stages (adult worm, metacercaria, cercaria and egg) were extracted by TRIzol methods and spectrophotometrically quantitated. Reverse transcription reactions were carried out to get the first-strand cDNA with the same quantity of total RNA as the template (1 µg). β-actin of *C. sinensis* (accession number: EU109284) was used as the transcription control. The real-time PCR amplification was performed using the LightCycler480 instrument (Roche, Switzerland) using the SYBR Premix ExTaq Kit. The LightCycler480 software (version 1.5) was used to analyze the data according to the 2^−ΔΔCt^ method [27]. The amplification of egg was employed as the calibrator to evaluate relative expression levels of *Cs*Pmy.

To analyze mRNA expression pattern of *Cs*Pmy in four developmental stages of *C. sinensis* including adult worm, metacercaria, cercaria and egg, we carried out qRT-PCR experiments with corresponding cDNA generated from total RNA. The results of qRT-PCR demonstrated *Cs*Pmy were expressed at the four examined stages (Figure 5). Normalized with *C. sinensis* β-actin, *Cs*Pmy transcribed highly at the stage of adult worm, metacercaria and cercaria while the expression level at egg was relatively low. Western blot analysis suggested *Cs*Pmy was translated among the four life stages with progressively increased expression level, which was accordance with qRT-PCR results (Figure 4B).

### Immunohistochemical localization of *Cs*Pmy at adult worm and metacercaria

**Figure 6:**
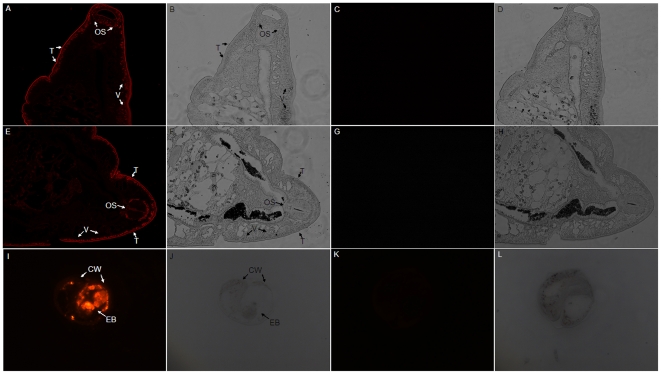
Immunohistochemical localization of *Cs*Pmy at adult worm and metacercaria. Adult worms and metacercariae of *C. sinensis* were fixed with 4% paraformaldehyde, embedded with paraffin and sliced into 3–5 µm in thick. The sections were blocked with normal goat serum overnight at 4°C, and then incubated with primary antibody (1∶200 dilutions) at room temperature for 2 h. After washing procedures, the sections were incubated with goat anti-rat IgG Alexa Fluor 594 (1∶400 dilutions) at room temperature for 1 h in dark. The images were captured under fluorescence microscope (ZEISS, Goettingen, Germany). Panel A–H, adult worm of *C. sinensis*. Pane I–L, metacercariae of *C. sinensis*. Pane A, B, E, F, I and J were sections treated with anti-pET26b-*Cs*Pmy serum. C, D, G, H, K and L were sections treated with naïve serum and imaged under the same conditions. Specific immunofluorescence was indicated in red (pane A, E and I), while no immunofluorescence was detected in pane C, G and K. Corresponding white light of parasite was panel B, D, F, H, J and L. **T**, tegument. **OS**, oral sucker. **V**, vitellarium. **CW**, cyst wall. **EB**, excretory bladder. Magnification for adult worm and metacercaria were ×100 and ×400, respectively.

In immunofluorescence assay (IFA), *Cs*Pmy was specifically localized at the tegument, oral sucker and vitellarium of adult worm (Figure 6, panel A and E). With the same dilution of anti-pET-26b-CsPmy rat serum, *Cs*Pmy was highly expressed at cyst wall and excretory bladder of metacercaria (Figure 6, panel I). While no specific fluorescence was detected in sections treated with naïve serum (Figure 6, panel C, G and K). Together, both IFA assay and above-mentioned immunoblot results confirmed the localization of *Cs*Pmy at the tegument of *C. sinensis*.

### Immune responses to *Cs*Pmy

**Figure 7:**
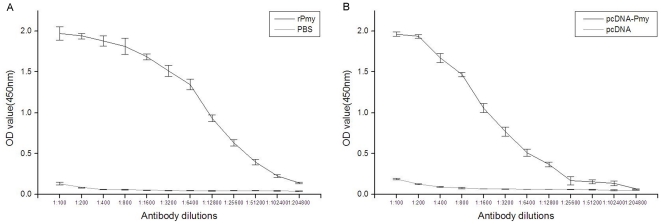
Antibody titers of IgG induced by *Cs*Pmy measured by ELISA. Briefly, 1 µg/well recombinant pET-26b-*Cs*Pmy protein was coated on the plates and blocked with 5% skimmed milk. The plate was incubated with different dilutions of the immune sera (week 6) raised by pET-26b-*Cs*Pmy and pcDNA-*Cs*Pmy. Rat sera immunized with PBS and pcDNA were measured under the same conditions as negative controls. HRP-conjugated IgG (1∶20000 dilutions) was used as the secondary antibodies. The reactions were developed with substrate solution TMB, stopped by 2 M H_2_SO4 and measured at measured at 450 nm. (A) Antibody titers of IgG induced by pET-26b-*Cs*Pmy. (B) Antibody titers of IgG induced by pcDNA-*Cs*Pmy.

**Figure 8:**
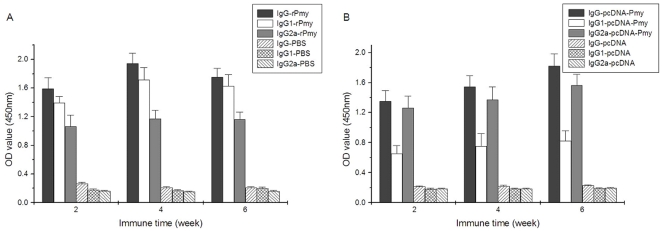
IgG isotype induced by *Cs*Pmy measured by ELISA. 1 µg/well recombinant pET-26b-*Cs*Pmy protein was coated on the plates and blocked with 5% skimmed milk. Immune sera from week 2 to week 6 were diluted at 1∶400. Rat sera immunized with PBS and pcDNA were measured under the same conditions as negative controls. IgG (1∶20000 dilutions), IgG1 and IgG2a (1∶1000 dilutions) were used as secondary antibodies. (A) Immune responses induced by pET-26b-*Cs*Pmy. (B) Immune responses induced by pcDNA-*Cs*Pmy.

As shown in Figure 7, we measured the antibody titers of total IgG in immunized sera of pET-26b-CsPmy group and pcDNA-*Cs*Pmy group. Antibody titers in the two groups peaked to 1∶204800 (Figure 7A) and 1∶102400 (Figure 7B), respectively, showing the high immunogenicity of *Cs*Pmy. After the first injection, serum level of IgG ascended rapidly both in pET-26b-*Cs*Pmy group (Figure 8A) and pcDNA-*Cs*Pmy group (Figure 8B). Additionally, we explored the Th1/Th2 type immune responses to *Cs*Pmy by measuring IgG1 and IgG2a from week 2 to week 6 post immunization (Figure 8). The ELISA results showed that combined Th1/Th2 immune responses were provoked by both pET-26b-*Cs*Pmy (Figure 8A) and pcDNA-*Cs*Pmy (Figure 8B) for both IgG1 and IgG2a level increased from week 2 to week 6. The increased IgG isotype demonstrated that combined cellular immunity and humoral immunity had been successfully induced by *Cs*Pmy.

### Protective effect of *Cs*Pmy in vaccination trials

The protective effect was assessed by comparing the worm burden and EPG among experimental groups. EPG was calculated three times from four weeks post challenge infection, all rats in four groups were sacrificed six weeks post infection. In Table 2, average worm numbers in pET-26b-*Cs*Pmy group (*n* = 6), PBS group (*n* = 6), pcDNA-*Cs*Pmy group (*n* = 6) and pcDNA group (*n* = 6) were 12.7, 27.8, 16.3, and 25.5, respectively. Average EPG in four groups was 1833.3, 3733.3, 2733.3, and 4466.7, respectively. Worm numbers and EPG in PBS group were significantly higher than those in pET-26b-*Cs*Pmy group (*p*<0.01, t = 9.524; *p*<0.01, t = 13.392). Meanwhile, worm numbers and EPG in pcDNA group were also significantly higher than those in pcDNA-*Cs*Pmy group (*p*<0.01, t = 13.216; *p*<0.01, t = 6.874). Worm reduction rate in pET-26b-*Cs*Pmy group and pcDNA-*Cs*Pmy group was 54.3% and 36.1%, respectively. The corresponding egg reduction rate was 50.9% and 38.8%, respectively. Comparing with the control groups, both pET-26b-*Cs*Pmy and pcDNA-*Cs*Pmy effectively lessened worm burden and EPG (see Table 2).

**Table 2:** Protective effect of in vaccination trials^a^.

Group	Worm burden	Worm reduction rate (%)	EPG	Egg reduction rate (%)
PBS (*n* = 6)	27.8±7.3		3733.3±467.6	
pcDNA (*n* = 6)	25.5±7.8		4466.7±1150.1	
rPmy (*n* = 6)	12.7±3.3**	54.30%	1833.3±542.8**	50.90%
pcDNA-Pmy (*n* = 6)	16.3±2.6**	36.10%	2733.3±546.5**	38.80%

a Protective effect was assessed by comparing the worm burden and EPG between pET-26b-*Cs*Pmy group and PBS group, as well as pcDNA-*Cs*Pmy group and pcDNA group, respectively. Results for analysis represented mean ± S.D., and the recovered worm numbers and EPG in groups were compared by Student's *t*-test.

(*) *p*<0.05 and.

(**) *p*<0.01 (compared to corresponding control).

## Discussion

The impact on public health of food-borne clonorchiasis is considerable since more than 35 million people are infected with *C. sinensis* and 601 million are at the risk of this neglected food-borne disease, which has been highly taken into account in regarding its serious complications [29]. Like other food-borne trematodes, vaccines and drugs are the two main interventions currently before the definite pathogenic mechanism are illuminated [9], [30]. The pressing demand for the development of novel vaccines of parasites relies on molecular and biological investigations on the crucial proteins, including serected and tegumental molecules [12], [15]. The present study explored the potential role of *C. sinensis* paramyosin as a vaccine candidate, which was identified from the cyst wall of *C. sinensis* metacercariae by HPLC-MS/MS. Cyst wall proteins of metacercariae and soluble tegumental components of adult worms were probed by anti-pET-26b-*Cs*Pmy immunized serum. *Cs*Pmy was expressed at four examined life stages and immunohistochemically localized at cyst wall and excretory bladder of metacercaria, and the tegument, oral sucker and vitellarium of adult worm. Immunoblot and immunolocalization results were consistent with previous discoveries that paramyosin was expressed on the tegument of *C. sinensis* [17], [18], [31], and were in supportive of our HPLC-MS/MS results that paramyosin was an important cyst wall protein of *C. sinensis* metacercariae. The proteomic approaches have provided us evidences of the specific localization and inspired us to further vaccine trials with *Cs*Pmy.

Recombinant pET-26b-*Cs*Pmy protein showed strong immunogenicity revealed by the rapidly increased IgG titer, which maintained for a high level from week 2 to week 6. In common with previous studies [32], we predicted 29 B-cell linear epitopes in the amino sequence of *Cs*Pmy, high level of antibody production may result from multiple B cell epitopes of paramyosin. DNA-based vaccine technology is a promising new tool in the development of vaccines to efficiently stimulate humoral (antibody) and cellular (T cell) immune responses to protein antigens, immunogenic proteins are expressed in *in vivo* transfected cells in their native conformation with correct posttranslational modifications [33]. DNA-based vaccines have been shown to confer immunity against different infectious diseases including parasitic infections based on their potential in inducing cellular immune responses [8], [34], [35]. Here, we investigated the protective efficacy of recombinant pcDNA-*Cs*Pmy plasmid which carried the full-length sequence of *Cs*Pmy. The strong immune responses elicited by pcDNA-*Cs*Pmy plasmid suggested that antigenic peptides of *Cs*Pmy were efficiently processed by antigen-presenting cells *in vivo*. As a sequence, both rats immunized with pET-26b-*Cs*Pmy protein and pcDNA-*Cs*Pmy plasmid exhibited strongly combined Th1/Th2 immune responses, which could be reflected by increased level of IgG1 and IgG2a in serum. We also evaluated immune responses by measuring Th1/Th2-associated cytokines level including IFN-γ and IL-4 (see Figure S2), which represented Th1 type and Th2 type immune responses [8], [34], [35]. Although increased level of IL-4 was lower than the level of IFN-γ in both pET-26b-*Cs*Pmy group and pcDNA-*Cs*Pmy group treated with TWE or rPmy, elevated IL-4 secretion was apparently induced by *Cs*Pmy, indicating that *Cs*Pmy was capable to induce combined cellular and humoral immunity (see Figure S2). To be expected, long-lasting strong antibody production resulted in significant reduction rate of worm burden and EPG in our preliminary vaccination trials, showing the potential of *Cs*Pmy as a *C. sinensis* vaccine.

In common with schistosomes, the tegument of *C. sinensis* is a dynamic host-interactive layer involved in nutrition ingestion, immune evasion, metabolin secretion, sensory reception and signal transduction [10]. Also, cyst wall of *C. sinensis* covers the entire body of metacercariae to protect juveniles from being attacked by immune response of host. In the present study, proteomic analysis enabled us to characterize the cyst wall proteins of *C. sinensis*, as a consequence, paramyosin was abundantly presented in cyst wall of metacercariae and tegument of adult worm. In addition, paramyosin was reported to exist predominantly in the tegument in non-filamentous form, this property is of particular importance in the design of new vaccines against a number of helminthes such as schistosomes [36], [37]. The immunomodulatory function of paramyosin during helminth infection and its surface localization have encouraged researchers to explore vaccine trials based on paramyosin [19], [38]. In our vaccine trials, *Cs*Pmy indeed exhibited protective effect against *C. sinensis* challenge and induced combined Th1/Th2 immune responses. Although the protective effect of paramyosin has been validated in a variety of trematodes and nematodes, the underlying mechanism remains to be clarified. Regarding the role of paramyosin in the biology of other parasites, it was suggested that protective effect of *Cs*Pmy could be resulted from the inhibitive effect of high level and long-lasting strong circulating antibodies on the muscle contraction and nutrition ingestion of *C. sinensis*. Furthermore, during the constantly contact with immune system of the host, paramyosin was also reported to perform non-muscular functions in host-parasite interactions by binding IgG, collagen and complement, which were related to the immune evasion of parasites [17], [39], [40]. Thus, further studies are required to break down the immune evasion of *C. sinensis* to enhance the current protective effect based on the physical and biological properties of *Cs*Pmy, as well as other identified cyst wall proteins.

Moreover, our studies indicated that full-length sequence of paramyosin was difficult to express in the usual prokaryotic systems. In order to obtain soluble *Cs*Pmy, we have tried various prokaryotic expressing vectors including pET-28a(+), pET-30a(+), pET-32a(+), pGEX-4T-1 and pQE-30 (see Figure S3). However, no recombinant protein was detected in *E. coli* using these expression plasmids (data not shown). Expression of *Cs*Pmy finally succeed in vector pET-26b(+) as inclusion bodies (Figure 3), the expression and purification of full-length sequence of *Cs*Pmy enabled us to evaluate paramyosin as a potential vaccine candidate as well as its biological properties in the future.

In conclusion, we have identified paramyosin from cyst wall of *C. sinensis* metacercariae by proteomic approaches and investigated the expression pattern in different life stages of *C. sinensis.* Moreover, we have explored the potential role of *Cs*Pmy as a protective vaccine candidate against *C. sinensis* infection. Furthermore, we developed Western blot analysis to investigate the antigenicity of *Cs*Pmy in *C. idellus* since *Cs*Pmy was identified from *C. sinensis* metacercariae which inhabited in *C. idellus*. As expected, *Cs*Pmy was probed by both the serum and the mucus of infected *C. idellus* (Figure S4). Coupled with our previous studies [41], vaccine trials with *Cs*Pmy carried out in rats encouraged us to develop vaccines in freshwater fish to combat cercarie infection by inhibiting cyst wall formation.

## Supporting Information

Figure S1 **Nucleotide sequences and amino acid sequences of** ***Cs*** **Pmy from our laboratory (** ***C. s-*** **1) and Korea (** ***C. s-*** **2).** The full-length complete sequence of *Cs*Pmy contains 3465 bp with an ORF (in red) of 2595 bp encoding 864 aa. Nucleotide and amino acid sequences described in the present study have been submitted to GenBank database under the accession number JQ041818. There are six base pairs and four amino acids differences between *C. s-*1 and *C. s-*2 (shaded in green). Five peptides (in blue) identified from HPLC-MS/MS matched with both *C. s-*1 and *C. s-*2 with the protein coverage of 6.5% (56/864). (A) Nucleotide sequence of *C. s-*1. (B) Amino acid sequence of *C. s-*1. (C) Nucleotide sequence of *C. s-*2. (D) Amino acid sequence of *C. s-*2.
(TIF)

Figure S2 **Cytokine production in spleen cells.** To evaluate cytokine production levels in immunized rats, the production of Th1/Th2-associated cytokines in splenocytes including Th1 type cytokine IFN-γ and Th2 type cytokine IL-4 were measured to evaluate the immune responses induced by *Cs*Pmy. Splenocytes were isolated from spleens of two rats in each group before challenge. The cells were washed three times with sterile PBS and treated with Erythrocyte Lysing Solution (Sigma) to remove red blood cells, then 5×10^5^ cells/well were cultured in 200 µl RPMI 1640 medium (Gibco, California, USA) supplemented with 10% FBS, 1% penicillin, and 1% streptomycin. Cytokine production of splenocytes was stimulated by TWE (50 µg/ml), rPmy protein (50 µg/ml), or medium alone as control. The 96-well plate (Corning, New York, USA) was maintained in an incubator at 37°C in 5% CO_2_ for 72 h. Cell-free supernatants were harvested and assayed for IFN-γ and IL-4 with ELISA kits (R&D Systems, Minneapolis, USA) according to the manufacturer's instruction. All assays were performed in duplicate. The concentration of IFN-γ and IL-4 calculated by using a linear-regression equation obtained from standard absorbance values. In contrast to control groups, both IFN-γ secretion (A) and IL-4 secretion (B) were induced by pET-26b-*Cs*Pmy and pcDNA-*Cs*Pmy when corresponding splenocytes were stimulated by TWE (*p*<0.01) or *Cs*Pmy (*p*<0.01).
(TIF)

Figure S3 **Recombinant plasmids of** ***Cs*** **Pmy in different prokaryotic expression vectors.** 1, 2 and 3 were PCR product of *Cs*Pmy, digestion of recombinant plasmid containing *Cs*Pmy and digestion of the corresponding blank plasmid, respectively. (A) Identification of recombinant pET-28a(+)-*Cs*Pmy with restriction enzymes. (B) Identification of recombinant pET-30a(+)-*Cs*Pmy with restriction enzymes. (C) Identification of recombinant pET-32a(+)-*Cs*Pmy with restriction enzymes. (D) Identification of recombinant pGEX-4T-1-*Cs*Pmy with restriction enzymes. (E) Identification of recombinant pQE-30-*Cs*Pmy with restriction enzymes. (F) Identification of recombinant pET-26b(+)-*Cs*Pmy with restriction enzymes.
(TIF)

Figure S4 **Immune responses to** ***Cs*** **Pmy in serum and mucus of** ***C. sinensis*** **-infected** ***C. idellus*** **.** As a cyst wall protein of metacercariae which dwell in freshwater fish, we investigated the antigenicity of *Cs*Pmy in the host *C. idellus*. (A) Western blot analysis of antigenicity. Briefly, the recombinant *Cs*Pmy (5 µg/lane) was subjected to SDS-PAGE (8% gel) and electrotransferred onto polyvinylidene difluoride (PVDF, Whatman, Maidstone, United Kingdom) membrane, the membrane was blocked with 5% (*w/v*) skim milk in phosphate buffered saline (PBS, pH 7.4) at 4°C overnight. The membrane was subsequently cut into strips then incubated with infected serum (1∶20 dilutions in 1% BSA-PBS) or undiluted infected mucus. Naïve serum and mucus were simultaneously incubated with the strips at room temperature for 2 h. Rabbit anti-fish HRP-conjugated secondary antibody (purchased from Chinese Academy of Medical Sciences) was reacted with strips in the dilution of 1∶1000 at room temperature for 2 h. Diaminobenzidine (DAB) substrate solution was used to visualize the reactions. As a result, we found that the recombinant *Cs*Pmy could probe serum (lane 1) and mucus (lane 3) from infected *C. idellus* while no reactions were found in naïve serum (lane 2) and mucus (lane 4). (B) ELISA assay of antibody titers. The infected serum, naïve serum, infected mucus and naïve mucus of *C. idellus* were gradiently diluted from 1∶1 to 1∶640, experiment protocols for ELISA were the same as described in Materials and Methods section. Although the level of circulated antibody was low and the immune response was relatively weak, ELISA assay showed antibody titers of IgM in serum and mucus reached to 1∶320, indicating that *Cs*Pmy could induce immune response in *C. idellus* which was the intermediate host.
(TIF)

Table S1 **Specific primers used in the present study.**
(DOC)
