# Supplementary figures and images for: Identification and Characterization of Paramyosin from Cyst Wall of Metacercariae Implicated Protective Efficacy against Clonorchis sinensis Infection
Source: PLoS One. 2012 Mar 21;7(3):e33703. doi: 10.1371/journal.pone.0033703 (PMC3312334; doi:10.1371/journal.pone.0033703)

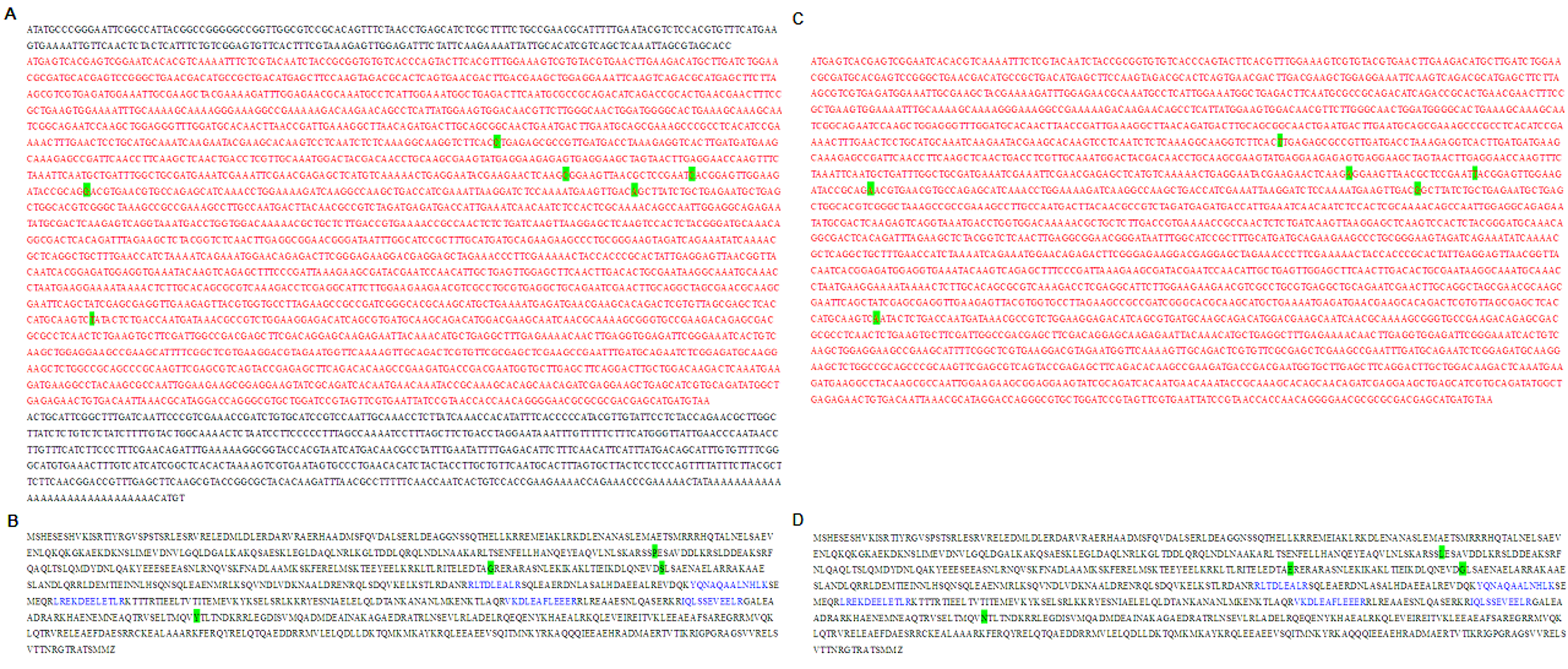

Supplement: Figure S1 — Nucleotide sequences and amino acid sequences of Cs Pmy from our laboratory ( C. s- 1) and Korea ( C. s- 2). The full-length complete sequence of CsPmy contains 3465 bp with an ORF (in red) of 2595 bp encoding 864 aa. Nucleotide and amino acid sequences described in the present study have been submitted to GenBank database under the accession number JQ041818. There are six base pairs and four amino acids differences between C. s-1 and C. s-2 (shaded in green). Five peptides (in blue) identified from HPLC-MS/MS matched with both C. s-1 and C. s-2 with the protein coverage of 6.5% (56/864). (A) Nucleotide sequence of C. s-1. (B) Amino acid sequence of C. s-1. (C) Nucleotide sequence of C. s-2. (D) Amino acid sequence of C. s-2. (TIF) [file pone.0033703.s001.tif]

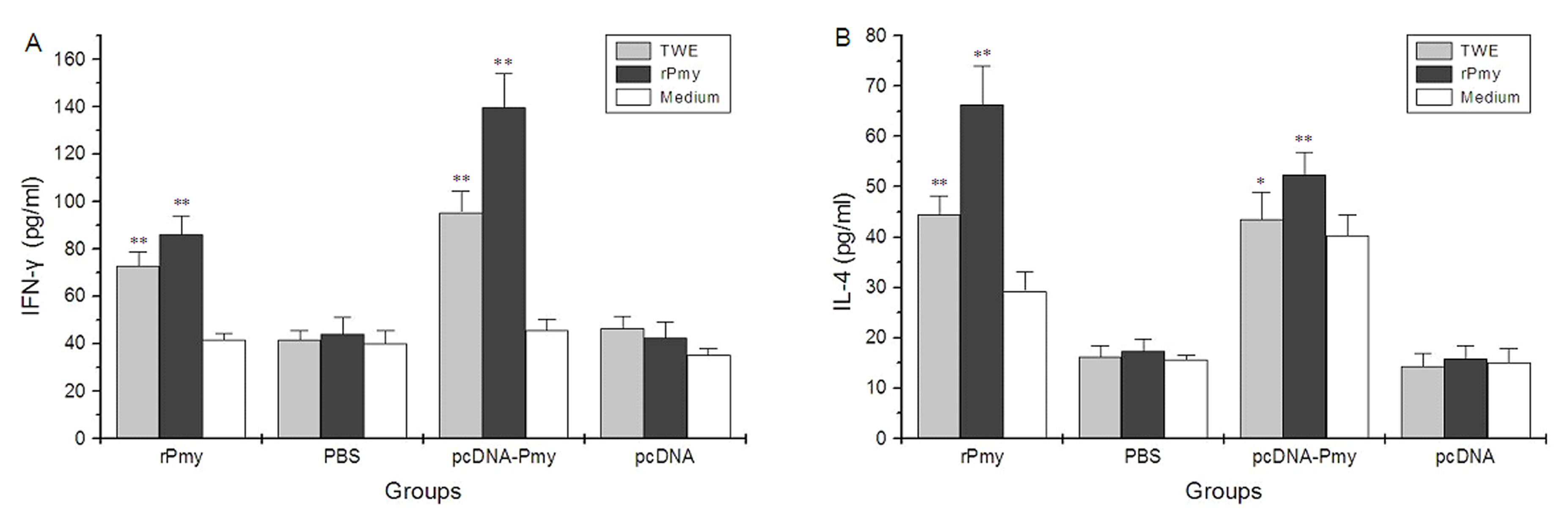

Supplement: Figure S2 — Cytokine production in spleen cells. To evaluate cytokine production levels in immunized rats, the production of Th1/Th2-associated cytokines in splenocytes including Th1 type cytokine IFN-γ and Th2 type cytokine IL-4 were measured to evaluate the immune responses induced by CsPmy. Splenocytes were isolated from spleens of two rats in each group before challenge. The cells were washed three times with sterile PBS and treated with Erythrocyte Lysing Solution (Sigma) to remove red blood cells, then 5×10^5^ cells/well were cultured in 200 µl RPMI 1640 medium (Gibco, California, USA) supplemented with 10% FBS, 1% penicillin, and 1% streptomycin. Cytokine production of splenocytes was stimulated by TWE (50 µg/ml), rPmy protein (50 µg/ml), or medium alone as control. The 96-well plate (Corning, New York, USA) was maintained in an incubator at 37°C in 5% CO2 for 72 h. Cell-free supernatants were harvested and assayed for IFN-γ and IL-4 with ELISA kits (R&D Systems, Minneapolis, USA) according to the manufacturer's instruction. All assays were performed in duplicate. The concentration of IFN-γ and IL-4 calculated by using a linear-regression equation obtained from standard absorbance values. In contrast to control groups, both IFN-γ secretion (A) and IL-4 secretion (B) were induced by pET-26b-CsPmy and pcDNA-CsPmy when corresponding splenocytes were stimulated by TWE (p<0.01) or CsPmy (p<0.01). (TIF) [file pone.0033703.s002.tif]

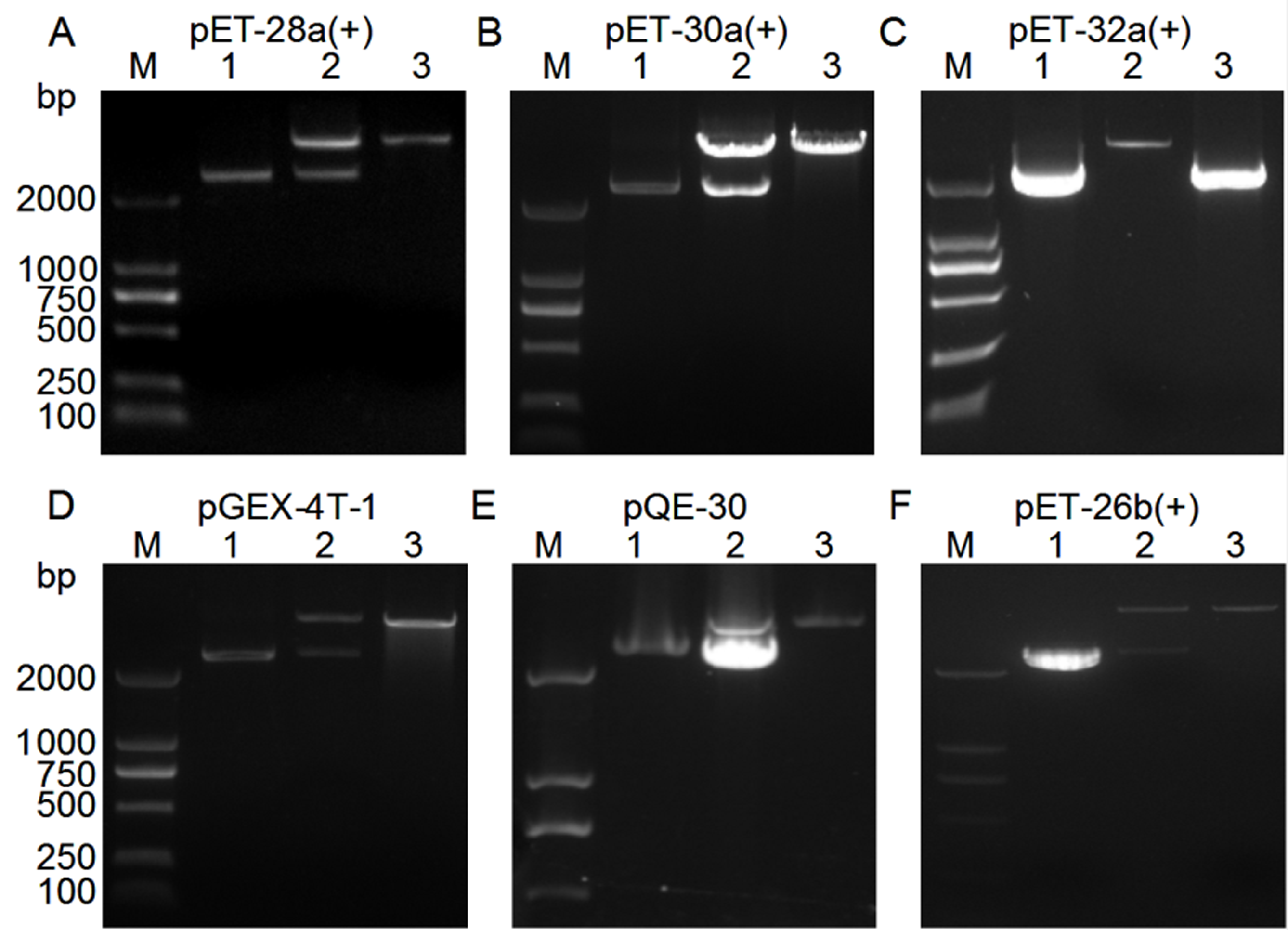

Supplement: Figure S3 — Recombinant plasmids of Cs Pmy in different prokaryotic expression vectors. 1, 2 and 3 were PCR product of CsPmy, digestion of recombinant plasmid containing CsPmy and digestion of the corresponding blank plasmid, respectively. (A) Identification of recombinant pET-28a(+)-CsPmy with restriction enzymes. (B) Identification of recombinant pET-30a(+)-CsPmy with restriction enzymes. (C) Identification of recombinant pET-32a(+)-CsPmy with restriction enzymes. (D) Identification of recombinant pGEX-4T-1-CsPmy with restriction enzymes. (E) Identification of recombinant pQE-30-CsPmy with restriction enzymes. (F) Identification of recombinant pET-26b(+)-CsPmy with restriction enzymes. (TIF) [file pone.0033703.s003.tif]

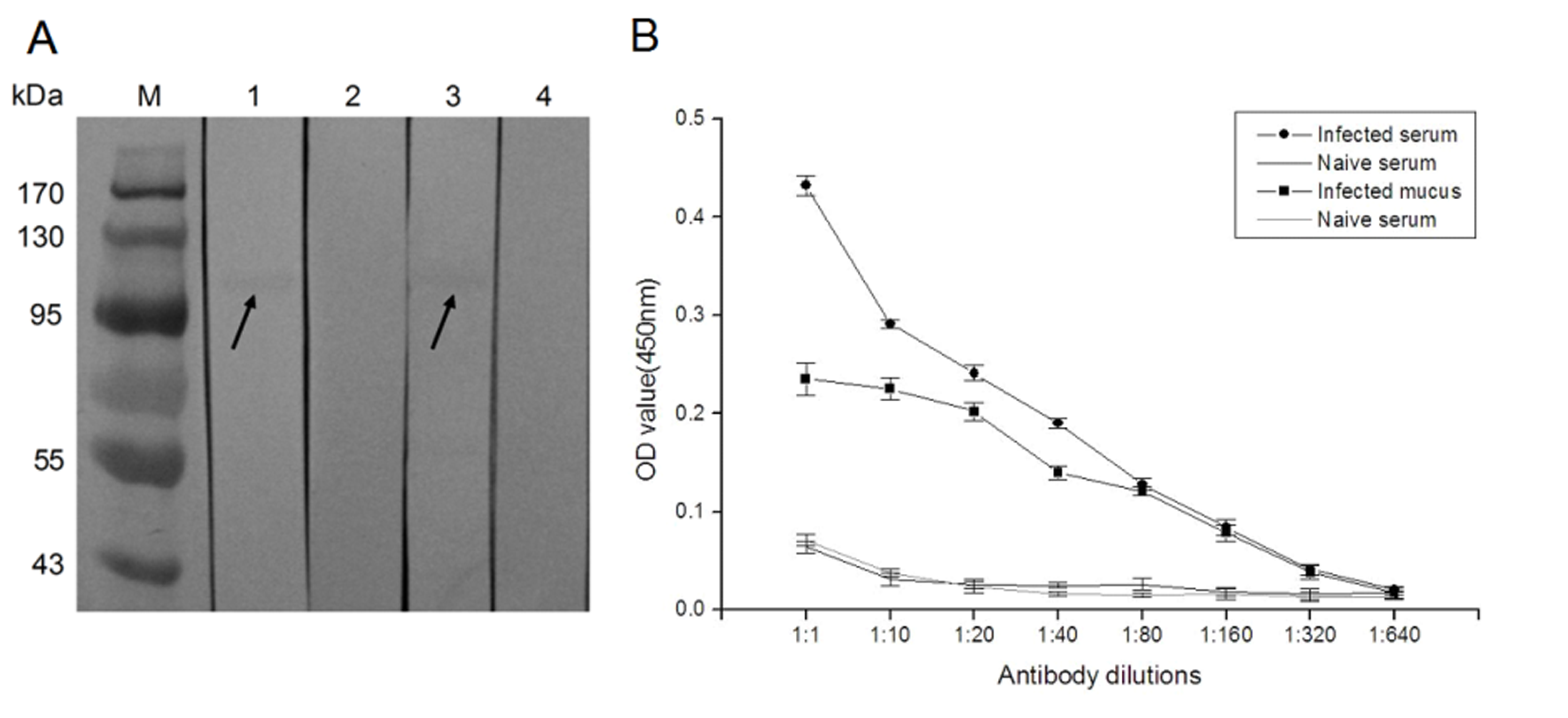

Supplement: Figure S4 — Immune responses to Cs Pmy in serum and mucus of C. sinensis -infected C. idellus . As a cyst wall protein of metacercariae which dwell in freshwater fish, we investigated the antigenicity of CsPmy in the host C. idellus. (A) Western blot analysis of antigenicity. Briefly, the recombinant CsPmy (5 µg/lane) was subjected to SDS-PAGE (8% gel) and electrotransferred onto polyvinylidene difluoride (PVDF, Whatman, Maidstone, United Kingdom) membrane, the membrane was blocked with 5% (w/v) skim milk in phosphate buffered saline (PBS, pH 7.4) at 4°C overnight. The membrane was subsequently cut into strips then incubated with infected serum (1∶20 dilutions in 1% BSA-PBS) or undiluted infected mucus. Naïve serum and mucus were simultaneously incubated with the strips at room temperature for 2 h. Rabbit anti-fish HRP-conjugated secondary antibody (purchased from Chinese Academy of Medical Sciences) was reacted with strips in the dilution of 1∶1000 at room temperature for 2 h. Diaminobenzidine (DAB) substrate solution was used to visualize the reactions. As a result, we found that the recombinant CsPmy could probe serum (lane 1) and mucus (lane 3) from infected C. idellus while no reactions were found in naïve serum (lane 2) and mucus (lane 4). (B) ELISA assay of antibody titers. The infected serum, naïve serum, infected mucus and naïve mucus of C. idellus were gradiently diluted from 1∶1 to 1∶640, experiment protocols for ELISA were the same as described in Materials and Methods section. Although the level of circulated antibody was low and the immune response was relatively weak, ELISA assay showed antibody titers of IgM in serum and mucus reached to 1∶320, indicating that CsPmy could induce immune response in C. idellus which was the intermediate host. (TIF) [file pone.0033703.s004.tif]
