# Supplementary material for: Identification and Characterization of Paramyosin from Cyst Wall of Metacercariae Implicated Protective Efficacy against Clonorchis sinensis Infection
Source: PLoS One. 2012 Mar 21;7(3):e33703. doi: 10.1371/journal.pone.0033703 (PMC3312334; doi:10.1371/journal.pone.0033703)
Supplement: Table S1 — Specific primers used in the present study. (DOC) [file pone.0033703.s005.doc]

Table S1. Specific primers used in the present study.

| Genes | Forward primers | Reverse primers |
| --- | --- | --- |
| pET-26b(+)-***Cs***Pmy | GTCCATATGATGAGTCACGAGTCGGAAT | ATAAAGCTTCATCATGCTCGTCGCG |
| pcDNA(+)-***Cs***Pmy | CTGAAGCTTATGAGTCACGAGTCGGAATC | GTCGATATCTTACATCATGCTCGTCGCG |
| pET-28a(+)-*Cs*Pmy | ATTCATATGATGAGTCACGAGTCGGAATC | ATAAAGCTTTTACATCATGCTCGTCGCG |
| pET-30a(+)-*Cs*Pmy | ATTCATATGATGAGTCACGAGTCGGAATC | ATAAAGCTTTTACATCATGCTCGTCGCG |
| pET-32a(+)-*Cs*Pmy | ATAGGTACCATGAGTCACGAGTCGGAAT | ATTAAGCTTTTACATCATGCTCGTCGCG |
| pGEX-4T-1-*Cs*Pmy | ATACCCGGGATGAGTCACGAGTCGGAAT | ATTGCGGCCGCTTACATCATGCTCGTCG |
| pQE-30-*Cs*Pmy | ATAGGTACCATGAGTCACGAGTCGGAAT | ATTAAGCTTTTACATCATGCTCGTCGCG |
| real-time PCR of ***Cs***Pmy | GGTTGAAGAGTTACGTGGTG | CGGCGTTTATCATTGGTC |
| real-time **PCR of *Cs***β-actin | GGTTGAAGAGTTACGTGGTG | CGGCGTTTATCATTGGTC |
